# Growth of a cohort of very low birth weight and preterm infants born at a single tertiary health care center in South Africa

**DOI:** 10.3389/fped.2022.1075645

**Published:** 2023-01-18

**Authors:** Isabel Alexandra Michaelis, Ingeborg Krägeloh-Mann, Mikateko Mazinu, Esme Jordaan

**Affiliations:** ^1^ Paediatric Department, Walter Sisulu University, Mthatha, South Africa; ^2^ University Children’s Hospital Tübingen, Tübingen, Germany; ^3^ Biostatistics Unit, South African Medical Research Council, Cape Town, South Africa

**Keywords:** VLBW (very low birth weight), preterm birth, growth, low-middle income country, low-resource setting, South Africa

## Abstract

**Background:**

Very low birth weight (VLBW) and extremely low birth weight (ELBW) infants are known to be at high risk of growth failure and developmental delay later in life. The majority of those infants are born in low and middle income countries.

**Aim:**

Growth monitoring in a cohort of infants born with a VLBW up to 18 months corrected age was conducted in a low resource setting tertiary hospital.

**Methods:**

In this prospective cohort study, 173 infants with a birth weight below 1,501 g admitted within their first 24 h of life were recruited and the 115 surviving until discharged were asked to follow up at 1, 3, 6, 12 and 18 months. Weight, height and head circumferences were recorded and plotted on WHO Z-score growth charts.

**Results:**

Of the 115 discharged infants 89 were followed up at any given follow-up point (1, 3, 6, 12 and/or 18 months). By 12 months of corrected age another 15 infants had demised (13.0%). The infants' trends in weight-for-age z-scores (WAZ) for corrected age was on average below the norm up to 12 months (average estimated z-score at 12 months = −0.44; 95% CI, −0.77 to −0.11), but had reached a normal range on average at 18 months = −0.24; 95% CI, −0.65 to 0.19) with no overall difference in WAZ scores weight between males and female' infants (*p* > 0.7). Similar results were seen for height at 12 months corrected age with height-for-age z-scores (HAZ) of the study subjects being within normal limits (−0.24; 95% CI, −0.63 to 0.14). The mean head circumference z-scores (HCZ) initially plotted below −1.5 standard deviations (S.D.), but after 6 months the z-scores were within normal limits (mean z-score at 7 months = −0.19; 95% CI, −0.45 to 0.06).

**Conclusion:**

Weight gain, length and head circumferences in infants with VLBW discharged showed a catch-up growth within the first 6–18 months of corrected age, with head circumference recovering best. This confirms findings in other studies on a global scale, which may be reassuring for health systems such as those in South Africa with a high burden of children born with low birth weights.

## Introduction

In the last few decades, the survival rate of preterm and very low birth weight (VLBW) infants has increased worldwide (1–3). However, while improved survival is a global trend, low- and middle-income countries (LMICs) are still lagging behind, as also shown in one of our previous studies of this cohort (3–6). Furthermore, post-discharge mortality of babies born with VLBW can be considerable in those countries (4, 7).

Minimizing the morbidity rate in surviving children has become a priority everywhere (1, 8–10). Infants born preterm or with a VLBW are at high risk of growth failure, developmental delays, neurological diseases like cerebral palsy (CP), and lower cognitive functioning in adulthood (11–20). Risk factors for postnatal growth failure in the preterm born child are male sex, small for gestational age, the need for mechanical or assisted ventilation, the use of postnatal steroids, sepsis and necrotising entero-colitis during their postnatal admission (9, 21–23). Preterm born infants also have a higher risk for cerebral palsy, which in its severe form is associated with poor growth, independent of prematurity (18, 19, 24, 25).

Weight gain, growth in length and, to a lesser extent, head circumference can be slower after birth in VLBW infants (7, 11, 26–28). Fortunately, in many parts of the world a good catch-up growth has been reported with head circumferences normalising within the first 3 months, while weight can take 6–12 months to reach the expected average z-score value. Normalization of height, however, is a concern mainly in LMICs (7, 11, 26–28).

Providing optimal nutrition and growth monitoring of preterm and VLBW infants during postnatal admission as well as post-discharge has been widely discussed and studied (29–32). Observational studies have suggested that rapid postnatal growth in preterm infants is linked to future development of metabolic diseases, whereas slow postnatal growth is associated with poorer neuro-cognitive outcomes (33–36). However, interventional studies have not shown such a clear relationship (27).

Data reporting on the postnatal growth of VLBW infants is limited in sub-Saharan Africa, despite the high rates of infants born with LBW or VLBW in this region (7, 28, 31, 37). The few studies available have demonstrated that postnatal catch-up growth in terms of weight is close to international standards, whilst stunting remains more prevalent (7, 28, 31, 37).

South Africa has been plagued by a double burden of malnutrition in recent years. The country is among the 24 countries that account for 80% of the world’s burden of stunting in children (38). In a cross-sectional in Limpopo, South Africa, Modjaji and Madida found that 22% of the 508 children studied were stunted and 27% were underweight, while 27% of their mothers were overweight and 42% were obese (38). The 2020 South African Child Gauge reported that 32% of children between 0 and 2 years of age were stunted and 5.9% were underweight (39).

Whilst challenging in under-resourced settings, postnatal growth monitoring is of utmost importance. It is cost-effective and can be done in a primary health care setting if the basic apparatuses are available and functioning (40). Plotting weight, height, and head circumferences is standard in any high risk infant follow-up clinic, although the charts used may differ (41–43). Monitoring postnatal growth allows to identify children that need intervention or more intensive follow-up monitoring.

### Setting

This study was conducted in a tertiary facility in the Eastern Cape Province, South Africa, which functions as a neonatal and paediatric referral center for surrounding primary health care clinics and district hospitals. Some referral centers may be further than 300 km with a challenging road infrastructure. The region belonged to the former homelands and is considered one of the most under-resourced areas of South Africa.

The public health sector cares mainly for people with very low socio-economic circumstances, even more so in the Eastern Cape, where 12.7% of its inhabitants live below the poverty line (southafrica-info.com).

The data collection is part of a South African Medical Research Council (SAMRC) funded prospective cohort study on outcome, growth and development of babies born with a birth weight of 1,500 g or less. A first publication is ‘Prospective cohort study of mortality in very low birthweight infants in a single centre in the Eastern Cape Province, South Africa” (6).

## Materials and methods

The study was approved by the institutional ethics committee (Walter Sisulu University Human Research Ethics Committee 086/2017). All infants born in the hospital or admitted within the first 24 h of life with a BW of ≤1,500 g were included (excluding infants with life-threatening congenital malformations). Recruitment lasted from December 2017 to November 2018, and mothers were approached for consent to include their data and participate in the follow-up at 1, 3, 6, 12, and 18 months of corrected age. Care-takers were not always able to come on the scheduled dates for various reasons.

At each follow-up, weight, height and head circumference were recorded as well as answers to routine questions concerning caregivers, type and amount of feeding and general well-being of the infant. The mothers or caregivers were asked to fill in the Ages and Stages Questionnaire (44, 45). Findings of the general and neurological examinations were recorded. Infants and toddlers were diagnosed with CP according to the definition by the SCPE and all children were classified according to the gross motor function classification system (GMFCS) (46–48). A full assessment on the neurodevelopmental outcome will be reported separately. Patients diagnosed with CP and a GFMCS level of IV or V at 12 and 18 months of corrected age were included in growth calculations, but also analyzed separately to determine whether these infants showed more severe failure to thrive according to the hypothesis that children with more severe CP show greater failure of growth, while children with lower levels of impairment grow similar to healthy preterm or VLBW infants. Furthermore, comparison of growth between appropriate for gestational age (AGA) and small for gestational age (SGA) infants, as well as between HIV exposed and HIV unexposed infants were separately analyzed.

If during consultation the need for auxiliary services was noted the infants were referred to the appropriate departments. All infants and children received standard care during this period. Breastfeeding support after discharge is not available in the hospital.

In total the mothers of 173 infants with a birth weight of ≤1,500 g were approached for inclusion in the study, of whom 161 infant-caregiver pairs consented. Forty-five of the infants died during admission and 115 were discharged with follow-up dates at 1, 3, 6, 12, and 18 months corrected age.

### Statistical analyses

The Fenton 2013 growth chart calculator was used to calculate the z score for babies at birth (49). The WHO Anthro program was employed to calculate the Z score of anthropometric variables of the follow-up periods, at 1, 3, 6, 12, and 18 months corrected age, obtaining the weight-for-age (WAZ), height-for-age (HAZ) and head circumference-for-age (HCZ) z-scores from the measured data. Categorical variables were described by frequencies and percentages, while continuous variables were described using mean (SD) or median (IQR).

A linear mixed (random coefficient) model was used to model the growth of the babies from 1 month to 18 months using WAZ, HAZ and HCZ. Age (corrected) was included as a continuous variable since babies were measured at irregular times at each visit (1, 3, 6, 12, 18 months), and age^2^ was also included to allow for a quadratic growth which were seen from the graphs for the growth of the babies. Gender was modeled as an interaction term to investigate growth for boys and girls separately. Random effects in the model were for the baby and the baby x age.

The growth of the 89 babies over time from 1 to 18 months and possible differences in growth between males and females were further investigated using a linear mixed model as described before. Growth was indicated as the mean differences in estimates from the previous age—the current age (e.g., mean difference at 3 months = 1 months - 3 month), so that a negative estimate indicates increased growth towards the normal range for the z value.

The weights between the different GMFCS groups were compared using the Kruskal-Wallis test, and to test for a difference between AGA and SGA infants for HIV exposed and unexposed infants an interaction term was entered into the mixed model.

## Results

**Figure 1:**
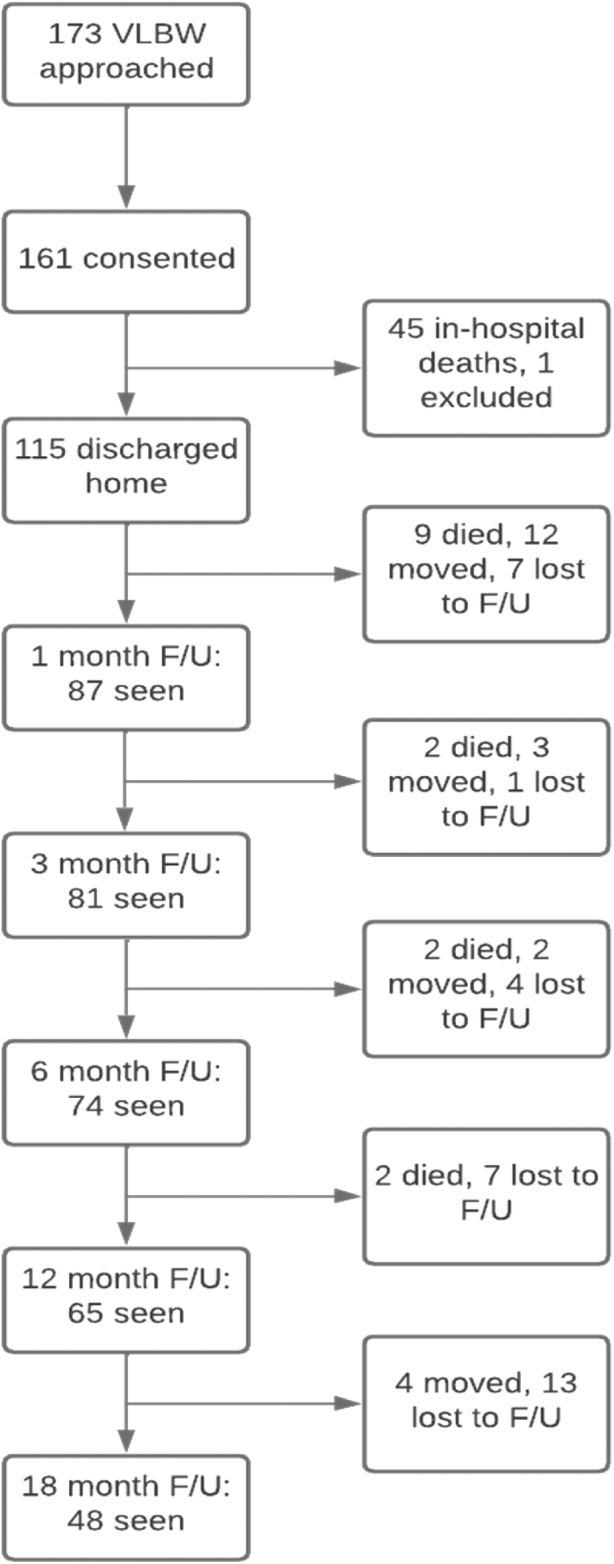
Flowchart of numbers of participants at each point of follow-up. One infant missed 1 and 3-months’, but attended 6–18 months’ follow-up.

Of the 115 infants discharged, 9 (7.8%) infants died at home within two weeks. Eighty-seven infants (75.7% of those discharged) attended their 1-month appointment. By 18-months 48 participants were followed-up (41.7% of those discharged), a total of 6 more children had died. See Figure 1. A total of 89 babies were seen at different times for their follow ups at 1, 3, 6, 12, and 18 months of corrected age. For their 1-month appointment, babies were seen between 0.3 and 1.7 months [mean (SD) = 0.9 (0.3)] and for their 18 months' appointment they were seen from 15.9 to 19.9 months [mean (SD) = 18.1 (0.8)]. See Figure 1. Descriptive and demographic data are summarized Table 1.

**Table 1:** Descriptive data of clinical and demographic variables.

	Birth	1 Month	3 Months	6 Months	12 Month	18 Months
Overall totals	115	87	81	74	65	48
	*n* (%)	*n* (%)	*n* (%)	*n* (%)	*n* (%)	*n* (%)
Lost to follow up						
Death	–	9 (32.1)	2 (25)	2 (25.0)	2 (20.0)	0 (0.0)
Moved	–	12 (42.9)	3 (37.5)	2 (25.0)	0 (0.0)	4 (26.7)
Other	–	7 (25.0)	3 (37.5)	4 (50.0)	8 (80.0)	11 (73.0)
Total	–	28 (100.0)	8 (100.0)	8 (100.0)	10 (100.0)	15 (100.0)
Gender						
Female	96 (59.6)					
Male	65 (40.4)					
Gestational week at birth						
25–32 GW	78 (49.1)					
33–37 GW	81 (50.9)					
Feeding option up to 6 months						
Breastfeeding	105 (91.3)	44 (50.6)	31 (38.3)	11 (15.0)	–	–
Formula	7 (6.1)	26 (29.9)	38 (46.9)	44 (59.0)	–	–
Mixed	3 (2.6)	17 (19.5)	12 (14.8)	19 (26.0)	–	–
Total	116 (100)	87 (100.0)	81 (100.0)	74 (100.0)	–	–
Feeding solids at 6 months						
Not yet	–	–	–	16 (21.9)	–	–
Yes	–	–	–	57 (78.1)	–	–
Total	–	–	–	73 (100.0)	–	–
Feeding 12 and 18months						
Well	–	–	–	–	60 (92.3)	47 (98.0)
Problems	–	–	–	–	5 (7.7)	1 (2.0)
Total	–	–	–	–	65 (100.0)	48 (100.0)
Primary care taker						
Parent		79 (91.8)	72(88.9)	68 (92.0)	60 (92.3)	43 (90.0)
Granny		4 (4.7)	3 (3.7)	3 (4.0)	3 (4.6)	4 (8.0)
Other		3 (3.5)	6 (7.4)	3 (4.0)	2 (3.1)	1 (2.0)
Total		86 (100.0)	81 (100.0)	74 (100.0)	65 (100.0)	48 (100.0)
HIV PCR						
Not exposed	71 (61.7)		54 (66.7)			36 (75)
Negative HIV test of exposed infants	44 (38.3)		27 (33.3)			12 (25)
Total	115 (100)		81 (100)			48 (100)
Size for GA at birth*						
Appropriate	18 (16)					
Small	95 (84)					

GA, gestational age; HIV PCR, Human immunodeficiency virus polymerase chain reaction.

^a^ Calculated using Fenton calculator.

A total of 89 babies were seen at different times for their follow-ups at 1, 3, 6, 12, and 18 months of corrected age. For their 1-month appointment, babies were seen between 0.3 and 1.7 months [mean (SD) = 0.9 (0.3)] and for their 18 months' appointment they were seen from 15.9 to 19.9 months [mean (SD) = 18.1 (0.8)].

### Clinical and demographic data

About half of the infants were born with a gestational age of 25–32 weeks (49.1%), the other half (50.9%) of 33–37 weeks (see Table 1). Common comorbidities during the hospital stay were congenital infections (tuberculosis, syphilis), hyaline membrane disease, neonatal jaundice, necrotizing enterocolitis, sepsis, and intraventricular hemorrhages.

The majority of infants were primarily cared for by their parent throughout the study (92%). During postnatal admission and according to the hospital guidelines almost all (91.3%) of the infants were exclusively breastfed, while only 6.1% were formula fed and 2.6% received mixed feeding (concurrent formula and breastfeeding). The proportion of infants who were exclusively breastfed declined steeply to 50.6% with a further 19.5% receiving mixed feeding at the corrected 1–month follow up. By 6 months, only 15% of infants were still exclusively breastfed The main reasons for cessation of breastfeeding was returning to work or the mother's concern of not having enough breastmilk for her baby (Table 2).

**Table 2:** Reason given for cessation of breastfeeding at 1 and 3 month's follow-up.

Discontinuation of EBF	Month 1 Number (%)	Month 3 Number & %
Insufficient breastmilk according to mother	10 (23%)	9 (43%)
Mom back to school/work or looking for work	15 (35%)	7 (33%)
Other (high HIV viral load, failing 2nd line HIV treatment, baby refusing breast according to mother, mother didn’t want to continue breastfeeding)	8 (19%)	3 (14%)
Unknown (no reason given)	10 (23%)	2 (10%)
Total	**43** **(100%)**	**21** **(100%)**

At discharge, 44 (38.2%) of the 115 alive infants were HIV-exposed, all of which had a negative HIV-PCR test at birth. At the 3-month follow-up, HIV-exposed infants had a second HIV-PCR test done, which at that time was negative in all HIV-exposed infants who came for follow-up. The recommended HIV ELISA at 18 months for exposed infants was negative for the remaining 12 infants. Analysis of growth difference between HIV exposed and HIV non-exposed infants showed no difference in their growth pattern. (see supplementary material).

Fifteen of the 89 (16.8%) patients were diagnosed with CP and 5 (5.6%) of those were classified into GMFCS level IV and V. We included all in the final analysis, but also analyzed them separately in two groups (GMFCS I-III and GMFCS IV- V) as indicated above. (see supplementary material).

A large proportion of discharged infants (84%) was born small for gestational age (SGA), as defined as BW below the 10th percentile for age and sex according to Fenton Preterm Growth Charts. All of the infants were born preterm (≤37 weeks of gestation). Analysis of growth difference between SGA and AGA infants showed no difference in their growth pattern (see supplementary material).

### Weight measurements from 1 to 18 months corrected age

The estimated average WAZ at birth could be extrapolated from the model and showed mean z score = −2.68 (95% CI, −2.98 to −2.38), which is well below −2SD.

**Figure 2:**
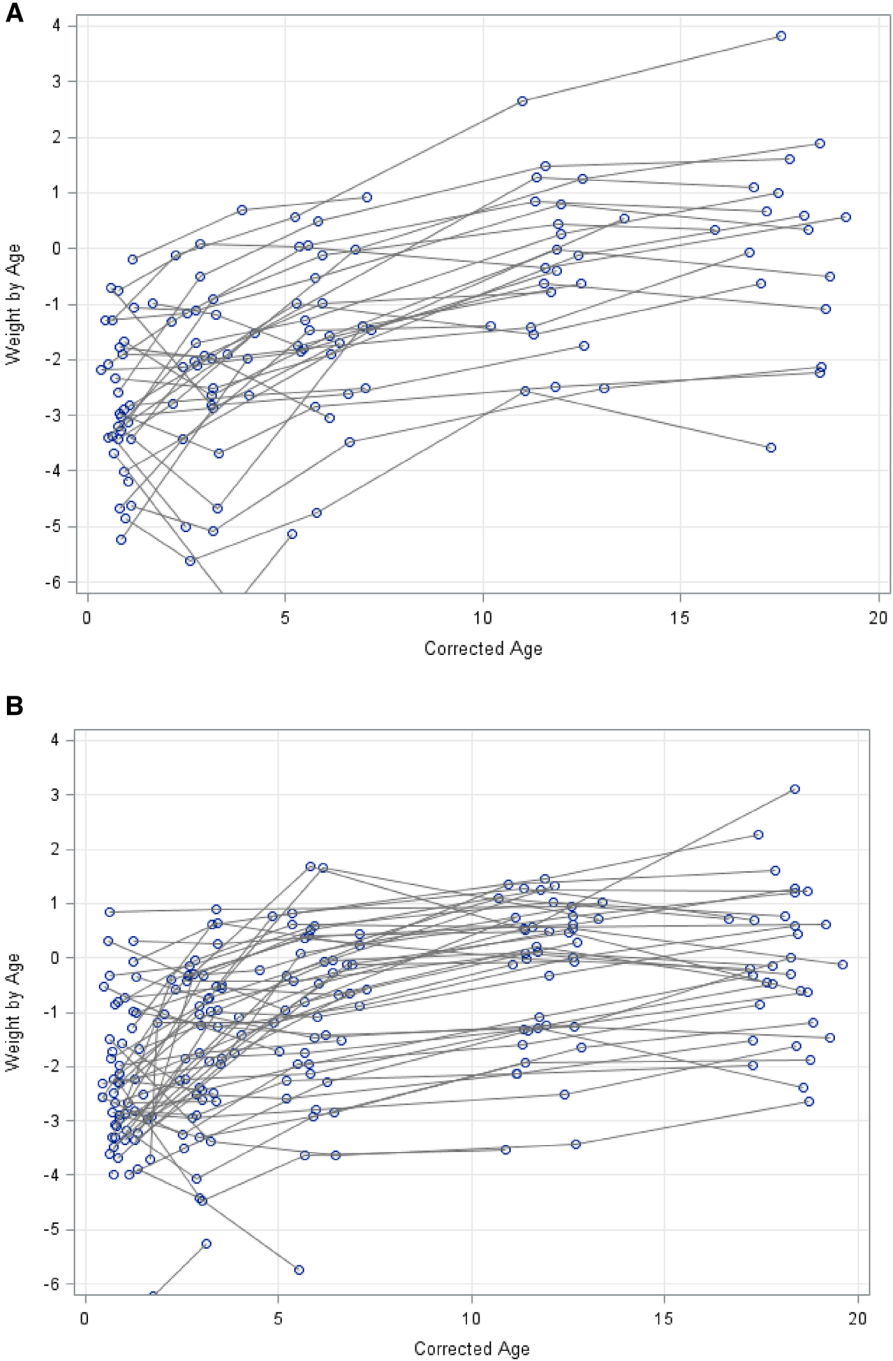
(**A**) growth weight-for-age z-scores in kg (WAZ) for males from 1 to 18 months. (**B**) growth weight-for-age Z-scores (WAZ) in kg for Females from 1 to 18 months.

The graphical representation of the raw data depicts the growth trajectory for each infant w.r.t. their weight for age z-scores (WAZ), showing that measurements at each time point were quite widely distributed around the time point, i.e., follow-up times were not exactly done at 1, 3, 6 and 18 months and not all babies attended each follow-up time. The growth curve followed a quadratic trend for both genders, with an accelerated growth from 1 to 12 months and then a levelling off towards 18 months (Figures 2A,B).

**Figure 3:**
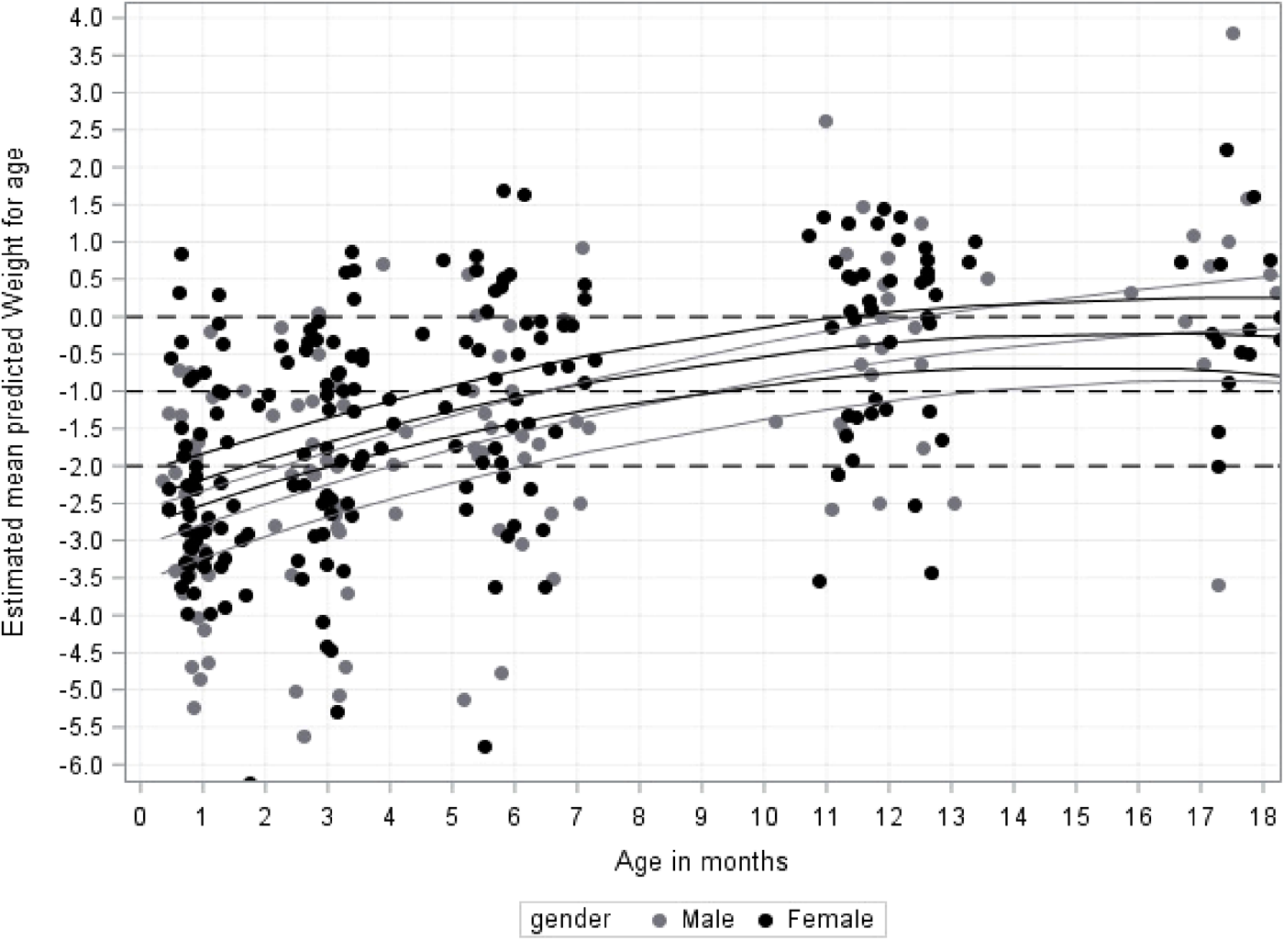
Growth for infant's (weight-for-age z-scores (WAZ) in kg from 1 to 18 months by gender.

The average WAZ values were below the norm from 1 month (average estimated z-score at 1 month = −2.4; 95% CI, −2.7 to −2.1) to 12 months (average estimated z-score at 12 months = −0.44; 95% CI, −0.77 to −0.11) with no overall difference between WAZ values for male and female infants (WAZ) (*p* > 0.7). However, some differences at individual time points were indicated between males and females, such as in the first 3 months marginally significant differences in WAZ between the sexes were found, with slightly lower values in males (*p* = 0.03). After 3 months the male infants' average WAZ values were comparable to the average WAZ scores for the females (Figure 3). Figure 3 also shows that the number of babies with a z-score of −2 or lower for WAZ gradually decreased from 1 month [58 (67%)] to 12 months [8 (12%)].

At the follow-up at 1 month corrected age the mean z-score for weight was below—2SD (−2.4; 95% CI, −2.68 to −2.12), and at the 6 months' follow-up the mean weight fell below −1 SD (−1.25; 95% CI, −1.54 to −0.97) for all assessed infants. At12 months corrected age the mean weight-for-age z-scores (WAZ) were within normal rage, (−0,439; 95% CI, −0.77 to −0.11) and at 18 months the mean WAZ age had improved further (−0.24; 95% CI, −0.65 to 0.18). Furthermore, at each check-up point between 1 and 12 months corrected age the mean weight increases significantly.

Children affected most severely by CP (GMFCS IV – V) showed a mean weight of 7.0 kg, compared to 8.1 kg and 9.4 kg (*p* = 0.005) for the GMFCS I-III, and to children not affected by CP at 12-months corrected age respectively. At 18-months corrected age the mean weight of the GMFCS IV – V group of 8.3 kg differed significantly to 9.8 kg and 11.0 kg (*p* = 0.009) of the two other groups (Table 3).

**Table 3:** Weight, height and head circumferences measurements between different cerebral palsy groups, compared with Kruskal Wallis test.

12 Month	GMFCS V and V	GMFCS I-III	No cerebral palsy	*p-*value
Weight	7.1 (6.8–7.5)	7.7 (7.3–9.1)	9.6 (8.8–10.2)	0.005
Height	68.0 (68.0–69.0)	71.5 (70.0–75.0)	74.8 (72.0–77.0)	0.009
Head circumference	43.1 (42.8–43.1)	44.3 (43.0–45.1)	45.5 (44.8–46.8)	0.025
18 Month				
Weight	8.0 (7.2–9.6)	9.1 (8.4–10.3)	11.0 (9.8–12.1)	0.009
Height	76.0 (69.0–78.5)	80.5 (76.0–81.5)	79.0 (78.0–81.5)	0.210
Head circumference	45.5 (42.5–45.5)	45.6 (44.7–46.1)	46.7 (45.6–48.7)	0.094

### Length/height measurements from 1 to 12 months corrected age

From the model below, the estimated average HAZ at birth could be extrapolated; z score = −3.38 (95% CI, −3.80 to −2.96), which was below −3SD.

**Figure 4:**
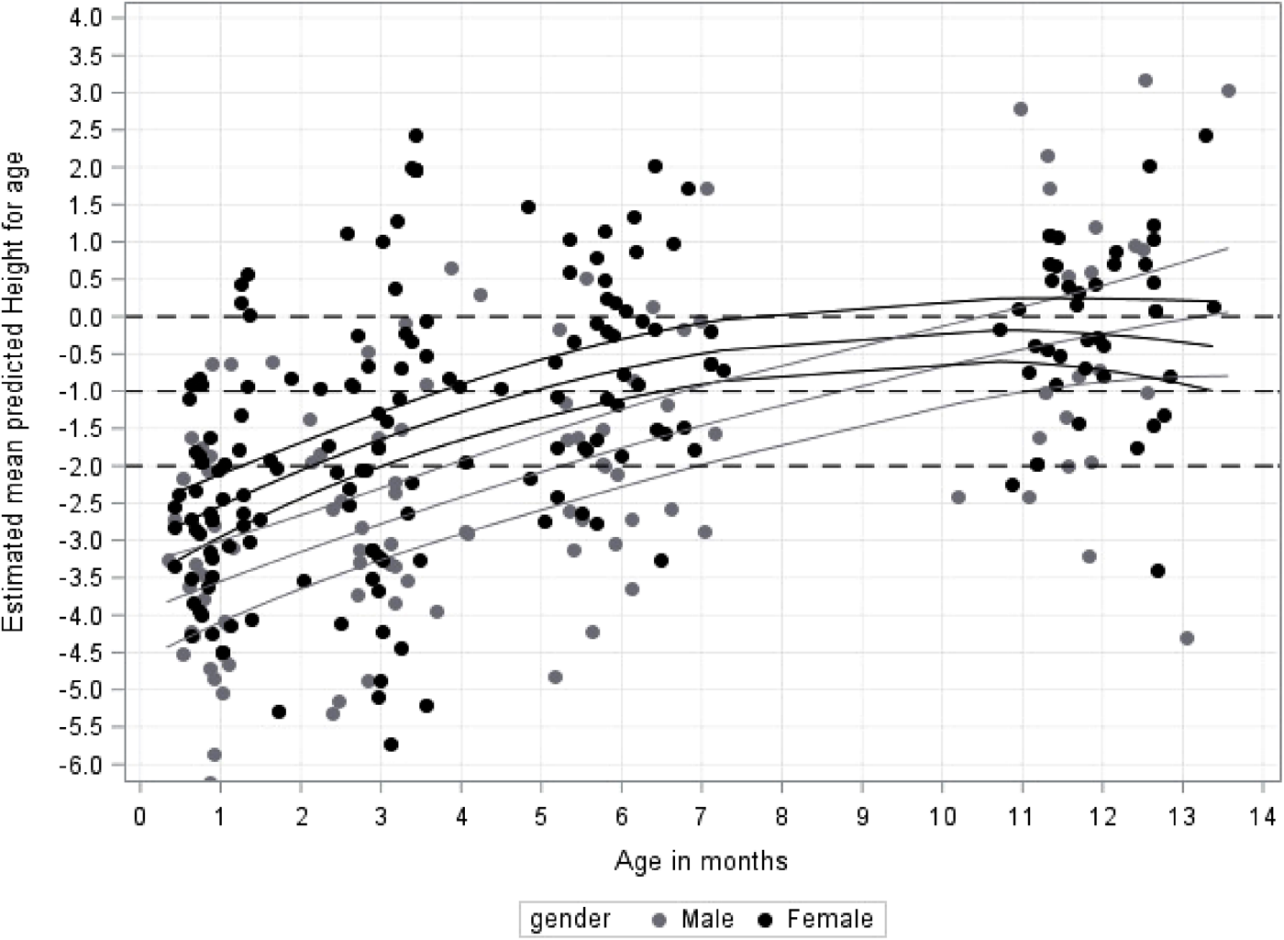
Growth for infant's (height-for-age z-scores) (HAZ) in cm from 1 to 12 months by gender.

Length and height measurements showed a similar trend as weight measurements in our study (Figure 4). Male infants started with a significant lower mean z-score for length for corrected age (HAZ) than female infants at 1 month (z-score difference = −1.0; 95% CI, −1.7 to −0.33; *p* = 0.004), 3 months (z-score difference = −1.1; 95% CI, −1.7 to −0.53; *p* = 0.0003) and 6 months (z-score difference = −1,1; 95% CI = −1.7 to −0.4; *p* = 0.002). At the 9 months' time point the male infants had caught up with the female infants in respect to the mean HAZ (z-score difference = −0.7; 95% CI, −1.34 to −0.009; *p* = 0.047). The mean increase in length and height was significant for all infants between each of the measurement points at 1–12 months, with values of −0.84 (SE = 0.09) (1 to 3 months), −0.96 (SE = 0.08) (3 to 6 months)) and −0.85 (SE = 0.19) (6 to 12 months) points at each 3-month interval, respectively (*p* = 0.0001). At 12 months the mean HAZ of the study subjects fell within normal limits (−0.24; 95% CI, −0.63 to 0.14). The 18 months' height data and comparisons were not included in our study as it showed a significant decrease of mean HAZ (*p* = 0.006), which is only explicable by continuous false measurement due to change in measurements taken with the infant lying (length) to standing (height).

Figure 4 also shows that the number of babies with a length or height of age below 2SDs gradually decreasing from 1 month [62 (71%)] to 12 months [7 (11%)].

Children affected most severely by CP (GMFCS IV – V) showed a mean length of 68.8 cm, compared to 72.0 and 75.0 cm (*p* = 0.009) for the GMFCS I-III and to children not affected by CP at 12 months corrected age, respectively (Table 3).

### Head circumference measurements from 1 to 18 months corrected age

**Figure 5:**
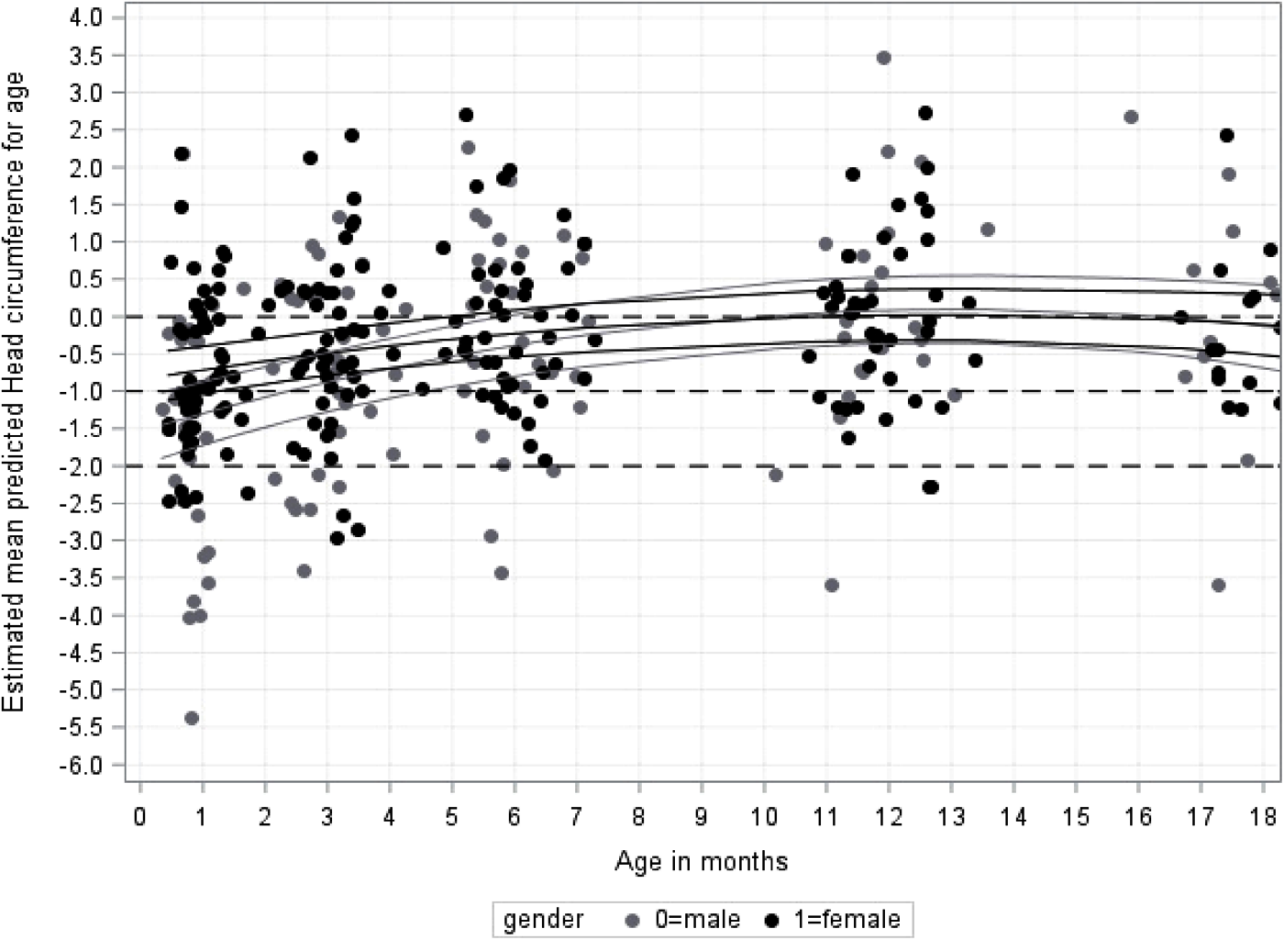
Growth for infant's head-circumference z-score for age (HCZ) in cm from 1 to 18 months by gender.

The male and female growth curves are provided in Figure 5. The lowest estimated average z-score for HCZ was at 1 month and was z-score = −0.9 (95% CI, −1.2 to −0.67). The average head circumferences for both sexes stayed below the 0 z-score for all time points up to 6 months (z-score at 6 months = −0.28; 95% CI, −0.53 to −0.03) and after 6 months the z-scores remained within normal limits.

A small difference in mean head circumferences for corrected age (HCZ) was seen between male and female infants at the 1month follow-up only (z-score difference = −0.58; 95% CI, −1.1 to −0.05; *p* = 0.031). Male infants caught-up to the females by 2 months of age (z-score difference = −0.48 (95% CI, −0.99 to 0.03; *p* = 0.063).

Figure 5 also shows that the number of babies below 2SDs gradually decreased from 1 month [16 (18%)] to 12 months [4 (6%)].

From the model, the estimated average HCZ at birth could be extrapolated; z-score = −1.10 (95% CI, −1.38 to −0.82), which is just below −1SD.

Children most affected by CP (GMFCS IV – V) had a mean HC of 43.0 cm compared to 44.3 and 45.7 cm (*p* = 0.025), for the GMFCS I-III and children unaffected by CP, respectively at 12-months follow-up, which was not significant (Table 3).

### Auxiliary services

At the corrected 1–month follow-up date, 28.7% of the infants had to be referred to the dietician due to insufficient growth and/or incorrect feeding practices. At the 6-months follow-up 43% of the children were referred to the dieticians, the number then declined to 12% at 12-months and 15% at the 18-months follow-up.

## Discussion

The survival rate of preterm and VLBW infants has increased globally (2, 50). Providing adequate post admission care and monitoring concerning growth and development is a significant challenge, especially in low- and middle-income countries, like in sub-Saharan Africa.

Our study confirmed the risk of VLBW dying after being discharged home (4, 7). Fifteen infants died before the 12 months' follow-up, most within the first month after discharge. This figure may be even higher as for approximately one-third of the patients the 12- and 18- month follow-ups fell within the period of strict lockdown in 2020 due to the COVID-Pandemic in South Africa and reasons for failure to attend are not known.

It is also important to know that the infants are discharged mostly into very poor social circumstances, with no further health care support for the mothers. Furthermore, about 20% of the mothers were less than 20 years of age at the time of birth and 85% were officially unemployed. Those circumstances make it difficult to care properly for those vulnerable infant to survive and thrive.

A very big concern is the high numbers of mothers who stopped breastfeeding and changed to formula feeds, sometimes days after discharge. Following the national guidelines of neonatal feeding, all infants had been receiving breastmilk during their neonatal stay unless there were medical contraindications. The two main reasons given was going back to school or work, looking for work or the perception, that the baby was not getting enough milk. This is especially worrying for this socio-economic group with very low income having to buy expensive formula feeds. This specific aspect of our cohort has been further explored and results concerning correct volume and preparation of the substitute feeds, as well as other socio-demographic relation will be available soon.

More than 80% of the babies discharged alive were SGA at birth using Fenton charts. This might be due to the high number of HIV exposed babies with most of their mothers on anti-retroviral medication, as well as the high number of mothers who had hypertension and/or preeclampsia (6). It was positive to see, however, that their growth restriction normalized over the next 12 months. No significant difference in their growth pattern was seen when compared to infants born with an AGA.

Our study could further confirm the findings that many preterm and VLBW infants struggle to gain weight and height appropriately, especially in their first year of life (5, 7, 11, 26, 31, 37). Weeks of failure of adequate growth is followed by a recovery of weight-for-age, length-for-age, and head circumferences by latest 12 months of corrected age. Similar to other studies, catch-up growth was most rapid for growth of head circumference, followed by weight, while length recovery lagged behind and remained lowest for all infants and both gender (5, 7, 40, 51). This trend is seen at birth already, with the head circumference for corrected age starting off with the values close to normal, while the weight for age z-scores were well below −2SD, and length-for-age below −3SD.

Some differences in growth were seen between male and female infants, with the males generally starting off with a lower mean weight and length, but catching up in all measurements at least by 6 months of corrected age. This has also been shown in other studies conducted in low-, and also high-income settings, even though complete catch-up to their female counterparts wasn’t always seen (5, 52, 53).

A concern is the relatively high loss to follow-up even though much effort was put into increasing compliance with remuneration and telephonic reminders. It would be important to look into the reasons to the reluctance of mothers to attend regular high-risk-follow-ups in order to improve them.

### Limitations

The study has several limitations. The main problem was the high loss of follow-up, which at 12- and 18 months was probably partly due to the COVID-19 lock-down which concerned about a third of the patient's appointment dates.

Other limitations of this study include the sample size limiting the ability to stratify or group by additional variables and measure their effects on growth patterns. However, this was not part of the primary aims of the study. Lastly, there were some limitations due to technical issues with the measurements for the height-for-age z-scores at 18 months, most likely arising from a continuous error of not making sure that the children standing in the correct upright position. Previous measurements were valid and could be included in the analyses.

## Conclusion

Infants born with a VLBW are vulnerable beyond their discharge as they are at a higher risk of death, growth and height deficits, as well as other health issues. In low-or middle-income countries preterm infants with ELBW or VLBW are often discharged earlier and with a lower weight into under-resourced homes which are often unable to provide suitable care for these infants. Furthermore, there is often insufficient support for the mothers or care-takers once they are discharged home, leading to a high percentage of further deaths after discharge.

Catch up growth in our study was similar to findings in other studies on a global scale, which may be reassuring for health systems such as those in South Africa with a high burden of children born with low birth weights and childhood malnutrition. Future research should explore the factors that lead to the high mortality post discharge and influences of the growth patterns, especially within the context of sub-Saharan Africa.

## Data Availability

The raw data supporting the conclusions of this article will be made available by the authors, without undue reservation.
